# Stage 2 Registered Report: Metacognitive asymmetries in visual perception

**DOI:** 10.1093/nc/niab025

**Published:** 2021-12-13

**Authors:** Matan Mazor, Rani Moran, Stephen M Fleming

**Affiliations:** Institute of Neurology, Wellcome Centre for Human Neuroimaging, University College London, London WC1N 3BG, UK; Institute of Neurology, Wellcome Centre for Human Neuroimaging, University College London, London WC1N 3BG, UK; Institute of Neurology, Max Planck UCL Centre for Computational Psychiatry and Ageing Research, London WC1B 5EH, UK; Institute of Neurology, Wellcome Centre for Human Neuroimaging, University College London, London WC1N 3BG, UK; Institute of Neurology, Max Planck UCL Centre for Computational Psychiatry and Ageing Research, London WC1B 5EH, UK; Department of Experimental Psychology, University College London, London WC1H 0AP, UK

**Keywords:** absence, presence, metacognition

## Abstract

Representing the absence of objects is psychologically demanding. People are slower, less confident and show lower metacognitive sensitivity (the alignment between subjective confidence and objective accuracy) when reporting the absence compared with presence of visual stimuli. However, what counts as a stimulus absence remains only loosely defined. In this Registered Report, we ask whether such processing asymmetries extend beyond the absence of whole objects to absences defined by stimulus features or expectation violations. Our pre-registered prediction was that differences in the processing of presence and absence reflect a default mode of reasoning: we assume an absence unless evidence is available to the contrary. We predicted asymmetries in response time, confidence, and metacognitive sensitivity in discriminating between stimulus categories that vary in the presence or absence of a distinguishing feature, or in their compliance with an expected default state. Using six pairs of stimuli in six experiments, we find evidence that the absence of local and global stimulus features gives rise to slower, less confident responses, similar to absences of entire stimuli. Contrary to our hypothesis, however, the presence or absence of a local feature has no effect on metacognitive sensitivity. Our results weigh against a proposal of a link between the detection metacognitive asymmetry and default reasoning, and are instead consistent with a low-level visual origin of metacognitive asymmetries for presence and absence.

## Introduction

At any given moment, there are many more things that are not there than things that are there. As a result, and in order to efficiently represent the environment, perceptual and cognitive systems have evolved to represent presences, and absence is implicitly represented as a default state (Oaksford and Chater 2001; Oaksford 2002). One corollary of this is that presence can be inferred from bottom-up sensory signals, but absence is never explicitly represented in sensory channels and must instead be inferred based on top-down expectations about the likelihood of detecting a hypothetical signal, had it been present. Experiments on human subjects accordingly suggest that representing absence is more cognitively demanding than representing presence, even in simple perceptual tasks, as is evident in slower reactions to stimulus absence than stimulus presence in near-threshold visual detection (Mazor *et al.* 2020), in a general difficulty to form associations with absence (Newman *et al.* 1980), and in the late acquisition of explicit representations of absence in development (e.g. Sainsbury 1971; Coldren and Haaf 2000; for a review on the representation of nothing, see Hearst 1991).

**Figure 1.**
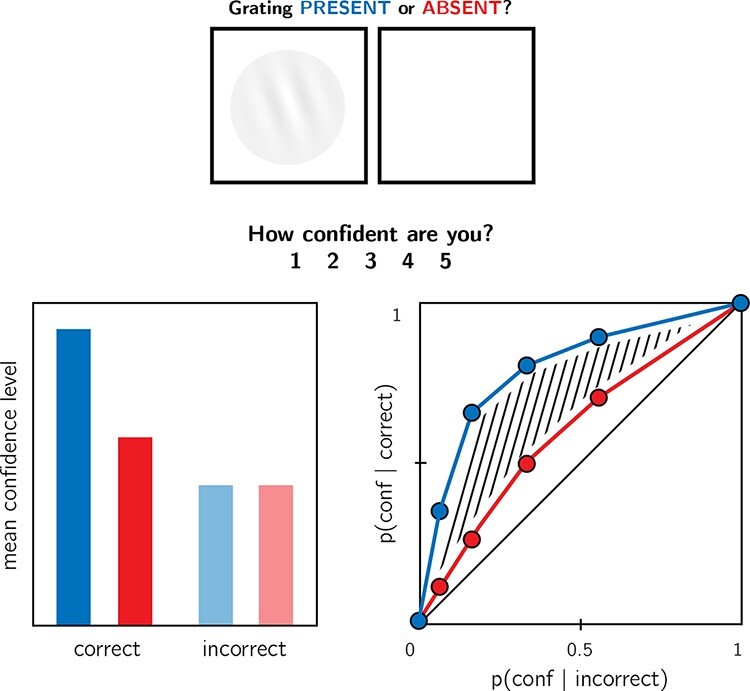
In visual detection, subjective confidence ratings following judgments about target absence are typically lower and less correlated with objective accuracy than following judgments about target presence. Top panel: a typical detection experiment. The participant reports whether a visual grating was present or absent and then rates their subjective decision confidence. Bottom left: typically, mean confidence in “yes” responses (blue) is higher than in “no” responses (red). This effect is much more pronounced in correct trials. Bottom right: the interaction between accuracy and response type on confidence (metacognitive asymmetry) manifests as a lower area under the response conditional type 2 Receiver Operating Characteristic (rcROC) curve for “no” responses compared with “yes” responses. Plots do not directly correspond to a specific dataset but portray typical results in visual detection

An overarching difficulty in representing absence may reflect the metacognitive nature of absence representations; to represent something as absent, one must assume that they would have detected it had it been present. In philosophical writings, this form of higher-order, metacognitive inference-about-absence is known as the ‘argument from epistemic closure’ or ‘argument from self-knowledge’ (“If it was true, I would have known it”; De Cornulier 1988; Walton 1992). Strikingly, quantitative measures of metacognitive insight are consistently found to be lower for decisions about absence than for decisions about presence. When asked to rate their subjective confidence following near-threshold detection decisions, subjective confidence ratings following “target absent” judgments are commonly lower and less aligned with objective accuracy, than following “target present” judgments (Fig. 1; Kanai *et al.* 2010; Kellij *et al.* 2021; Mazor *et al.* 2020; Meuwese *et al.* 2014).

Metacognitive asymmetries have not only been observed for judgments about the presence or absence of whole physical objects and stimuli but also for the presence or absence of cognitive variables such as memory traces. For instance, in recognition memory, subjects typically show poor metacognitive sensitivity for judgments about the absence of memories (such as when judging that they have not seen a study item before; Higham *et al.* 2009). Unlike the absence of a visual stimulus, the absence of a memory is not localized in space and does not correspond with a specific representation of “nothing.”

One way of conceptualizing these findings is that absence asymmetries emerge as a function of default reasoning—absences are considered the *default,* and information about perceptual or mnemonic presence is accumulated and tested against this default. For instance, an asymmetry may emerge in recognition memory because the presence of memories is actively represented, and the absence of memories is assumed as the default unless evidence is available for the contrary. In the same way, other visual features that are not typically treated as presences or absences may still be coded relative to a default, assuming one state unless evidence is available for the contrary (e.g. assuming that a cookie is sweet rather than salty). However, whether a metacognitive asymmetry in processing presence and absence generalizes to these more abstract violations of default expectations remains unknown. Here, we set out to map out the structure of absence representations by testing for metacognitive asymmetries in the presence and absence of attributes at different levels of representation—from concrete objects, to visual features, to violations of default expectations.

Our choice of stimuli draws inspiration from visual search—a field where asymmetries are observed for a variety of stimulus types and features. In visual search, participants typically take longer to search for a target that is marked by the absence of a distinguishing feature, as compared to searching for a target that is marked by the presence of a feature relative to distractors (Treisman and Souther 1985; Treisman and Gormican 1988). Interestingly, *search asymmetries* have been demonstrated not only for the absence or presence of concrete physical features but also for the presence or absence of deviations from a more abstract default state, which can be based on experience, culture, and contextual expectations (see the Methods section; Frith 1974; Von Grünau and Dubé 1994; Wang *et al.* 1994; Gandolfo and Downing 2020). Of special interest for our study are these latter asymmetries due to expectation violations and their relation with asymmetries induced by the presence or absence of local and global features. Observing a metacognitive asymmetry for expectation violations as well as for the presence and absence of object features would support a strong link between the representation of absence and default reasoning, where differences in metacognitive sensitivity reflect differences in the processing of information that agrees or contrasts with the expected default state.

 While traditional accounts interpreted visual search asymmetries as reflecting a qualitative advantage for the cognitive representation of presence (affording a parallel search in the case of feature-present search only; Treisman and Gormican 1988), other models attribute the asymmetry to differences in the distributions of perceptual signals already at the sensory level (Dosher *et al.* 2004; Vincent 2011). Similarly, in the case of metacognitive asymmetries, the idea that decisions about absence are qualitatively different from decisions about presence has been challenged by an excellent fit of simple models that assume unequal variance for the signal-present and signal-absent sensory distributions, a model that does not assume any qualitative difference between the two decisions (Kellij *et al.* 2021). Deciding between these model families is beyond the scope of this project. However, identifying metacognitive asymmetries for abstract cognitive variables such as familiarity could help refine these models, for instance, by revealing that representing deviations from a default state is an overarching principle of cognitive organization, one that goes beyond specific features of visual perception.

## Methods

We report how we determined our sample size, all data exclusions (if any), all manipulations, and all measures in the study. The full registered protocol is available at osf.io/ed8n7.

**Figure 2.**
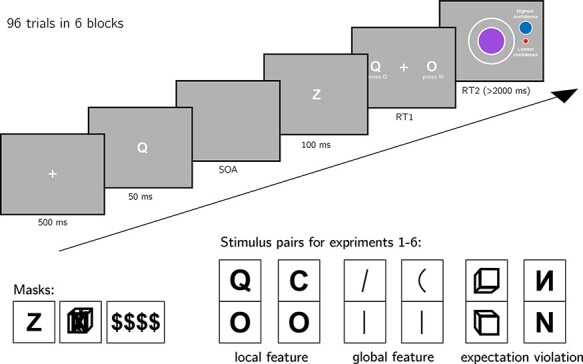
Experiment design. Metacognitive asymmetry effects were tested for six stimulus features in six separate experiments, encompassing three levels of abstraction: local features, global features, and expectation violations. The presented trial corresponds to the first stimulus pair, with *Q* and *O* as the two stimuli

We ran six experiments that were identical except for the identity of the two stimuli ${S_1}$ and ${S_2}$ (and of the stimulus used for backward masking; see the “Deviations from pre-registration” section for details). Our choice of stimuli for this study was based on the visual search literature. For some stimulus pairs ${S_1}$ and ${S_2}$, searching for one ${S_1}$ among multiple ${S_2}$s is more efficient than searching for one ${S_2}$ among multiple ${S_1}$s. Such *search asymmetries* have been reported for stimulus pairs that are identical except for the presence and absence of a distinguishing feature. Importantly, distinguishing features vary in their level of abstraction, from concrete *local features* (finding a *Q* among *O*s is easier than the inverse search; Treisman and Souther 1985), through *global features* (finding a curved line among straight lines is easier than the inverse search; Treisman and Gormican 1988), and up to the presence or absence of abstract *expectation violations* (searching for an upward-tilted cube among downward-tilted cubes is easier than the inverse search, in line with a general expectation to see objects on the ground rather than floating in space; Von Grünau and Dubé 1994). We treat these three types of asymmetries as reflecting a default-reasoning mode of representation, where the absence of features and/or the adherence of objects to prior expectations is tentatively accepted as a default by the visual system, unless evidence is available for the contrary (Treisman and Souther 1985; Treisman and Gormican 1988). In this study, we test for metacognitive asymmetries for two stimulus features in each category, in six separate experiments with different participants (Fig. 2). For each of the following stimulus pairs, searching for ${S_1}$ among multiple instances of ${S_2}$ has been found to be more efficient than the inverse search:

- **Local feature: Addition of a stimulus part.** *Q* and *O* were used as ${S_1}$ and ${S_2}$, respectively (Treisman and Souther 1985).
- **Local feature: Open ends.** *C* and *O* were used as ${S_1}$ and ${S_2}$, respectively (Treisman and Souther 1985; Treisman and Gormican 1988; Takeda and Yagi 2000).
- **Global feature: Orientation.** Tilted and vertical lines were used as ${S_1}$ and ${S_2}$, respectively (Treisman and Gormican 1988).
- **Global feature: Curvature**. Curved and straight lines were used as ${S_1}$ and ${S_2}$, respectively (Treisman and Gormican 1988).
- **Expectation violation: Viewing angle**. Upward- and downward-tilted cubes were used as ${S_1}$ and ${S_2}$, respectively (Von Grünau and Dubé 1994).
- **Expectation violation: Letter inversion.** Flipped and normal *N* were used as ${S_1}$ and ${S_2}$, respectively (Frith 1974; Wang *et al.* 1994).

The experiments quantified participants’ metacognitive sensitivity for discrimination judgments between ${S_1}$ and ${S_2}$.

### Participants

The research complied with all relevant ethical regulations and was approved by the Research Ethics Committee of University College London (study ID number 1260/003). Participants were recruited via Prolific and gave informed consent prior to their participation. They were selected based on their acceptance rate (>95%) and for being native English speakers. For each of the six experiments, we aimed to collect data until we reached 106 included participants (after applying our pre-registered exclusion criteria). The entire experiment took 10–15 minutes to complete. Participants were paid between £1.25 and £2 for their participation, maintaining a median hourly wage of £6 or higher.

### Procedure

Experiments were programmed using the jsPsych and P5 JavaScript packages (De Leeuw 2015; McCarthy 2015) and were hosted on a JATOS server (Lange *et al.* 2015).

After instructions, a practice phase, and a multiple-choice comprehension check, the main part of the experiment started. It comprised 96 trials separated into 6 blocks. Only the last five blocks were analyzed.

On each trial, participants made discrimination judgments on masked stimuli and rated their subjective decision confidence on a continuous scale. After a fixation cross (500 ms), the target stimulus (${S_1}$ or ${S_2}$) was presented in the center of the screen for 50 ms, followed by a mask (100 ms). Stimulus Onset Asynchrony (SOA) was calibrated online in a 1-up-2-down procedure (Levitt 1971), with a multiplicative step factor of 0.9 and starting at 30 ms. Participants then used their keyboard to make a discrimination judgment. Stimulus-key mapping was counterbalanced between participants. Following responses, subjective confidence ratings were given on an analog scale by controlling the size of a colored circle with the computer mouse. High confidence was mapped to a big, blue circle, and low confidence to a small, red circle. We chose a continuous (rather than a more typical discrete) confidence scale in order to ensure sufficient variation in confidence ratings within the dynamic range of individual participants. This variation is useful for the extraction of response conditional type 2 Receiver Operating Characteristic (ROC) curves. The confidence rating phase terminated once participants clicked their mouse but not before 2000 ms. No trial-specific feedback was delivered about accuracy. In order to keep participants motivated and engaged, block-wise feedback was delivered between experimental blocks about overall accuracy, mean confidence in correct responses, and mean confidence in incorrect responses. Online demos of the experiments can be accessed at matanmazor.github.io/asymmetry.

#### Randomization

The order and timing of experimental events were determined pseudo-randomly by the Mersenne Twister pseudorandom number generator, initialized in a way that ensures registration time-locking (Mazor *et al.* 2019).

### Data analysis

We used R (Version 3.6.0; R Core Team 2019) and the R-packages *BayesFactor* (Version 0.9.12.4.2; Morey and Rouder 2018), *broom* (Version 0.5.6; Robinson and Hayes 2020), *cowplot* (Version 1.0.0; Wilke 2019), *dplyr* (Version 1.0.4; Wickham *et al.* 2020), *ggplot2* (Version 3.3.1; Wickham 2016), *lmerTest* (Version 3.1.2; Kuznetsova *et al.* 2017), *lsr* (Version 0.5; Navarro 2015), *MESS* (Version 0.5.6; Ekstrøm 2019), *papaja* (Version 0.1.0.9997; Aust and Barth 2020), *pracma* (Version 2.2.9; Borchers 2019), *pwr* (Version 1.3.0; Champely 2020), and *tidyr* (Version 1.1.0; Wickham and Henry 2020) for all our analyses.

For each of the six stimulus pairs [${S_1}$, ${S_2}$], we tested the following hypotheses:

1. *Hypothesis 1:* Subjective confidence is higher for ${S_1}$ responses than for ${S_2}$ responses.

For each of the six stimulus pairs, we tested the null hypothesis that subjective confidence for ${S_1}$ responses is equal to or lower than subjective confidence for the *S*_2_ responses (${H_o}:con{f_{{S_1}}} \le con{f_{{S_2}}}$).

1. *Hypothesis 2:* Metacognitive sensitivity, measured as the area under the response conditional type 2 ROC curve, is higher for ${S_1}$ responses than for ${S_2}$ responses.

For each of the six stimulus pairs, we tested the null hypothesis that metacognitive sensitivity for ${S_1}$ responses is equal to or lower than metacognitive sensitivity for ${S_2}$ responses (${H_o}:auRO{C_{{S_1}}} \le auRO{C_{{S_2}}}$).

1. *Hypothesis 3:* Metacognitive sensitivity, measured as the area under the response conditional type 2 ROC curve, is higher for ${S_1}$ responses than for ${S_2}$ responses, to a greater extent than expected from an equivalent equal-variance Signal Detection Theory (SDT) model.

For each of the six stimulus pairs, we tested the null hypothesis that difference between metacognitive sensitivities for ${S_1}$ and ${S_2}$ responses is lower than the difference expected from an equivalent equal-variance SDT model (${H_o}:\left( {auRO{C_{{S_1}}} - auRO{C_{{S_2}}}} \right) \le \left( {{{\widehat {auROC}}_{{S_1}}} - {{\widehat {auROC}}_{{S_2}}}} \right)$ where $\widehat {auROC}$ is the expected area under the rc-ROC curve (auROC_2_) under an equal variance SDT model with equal sensitivity, criterion, and distribution of confidence ratings in incorrect responses).

1. *Hypothesis 4:* ${S_1}$ responses are faster on average than ${S_2}$ responses.

For each of the six stimulus pairs, we tested the null hypothesis that log-transformed response times for ${S_1}$ responses are equal to or higher than log-transformed response times for ${S_2}$ responses (${H_o}:log\left( {R{T_{{S_1}}}} \right) \ge log\left( {R{T_{{S_2}}}} \right)$).

Hypotheses 1 and 2 correspond to the effects of stimulus type on metacognitive bias and metacognitive sensitivity, respectively. Although these two measures are theoretically independent, both bias and sensitivity are found to vary between detection “yes” and “no” responses.

Based on pilot data and previous experiments examining near-threshold perceptual detection and discrimination, we did not expect a response bias (such that the probability of responding ${S_1}$ is significantly different from 0.5 across participants). However, such a response bias, if found, may bias metacognitive asymmetry estimates as measured with response conditional type 2 ROC curves. Hypothesis 3 was designed to confirm that metacognitive asymmetry is higher than that expected from an equivalent equal-variance SDT model with the same response bias, sensitivity, and distribution of confidence ratings in incorrect responses as in the actual data. We interpreted conflicting results for Hypotheses 2 and 3 as evidence for a metacognitive asymmetry that is driven or masked by a response bias.

Hypothesis 4 is motivated by two observations from previous studies. First, detection “yes” responses are faster than detection “no” responses (Mazor *et al.* 2020). Second, when participants are not under strict time pressure, reaction time inversely scales with confidence (Henmon 1911; Pleskac and Busemeyer 2010; Moran *et al.* 2015; Calder-Travis *et al.* 2020). Based on these findings, if ${S_1}$ and ${S_2}$ responses are similar to detection “yes” and “no” responses not only in explicit confidence judgments but also in response times, we should also expect a response time difference for these stimulus pairs.

#### Dependent variables and analysis plan

Response conditional type 2 ROC curves were extracted by plotting the empirical cumulative distribution of confidence ratings for correct responses against the same cumulative distribution for incorrect responses. This was done separately for the two responses ${S_1}$ and ${S_2}$, resulting in two curves. The area under the response conditional type 2 ROC curve is a measure of metacognitive sensitivity (Fleming and Lau 2014). The difference between the areas for the two responses is a measure of metacognitive asymmetry (Meuwese *et al.* 2014). This difference was used to test Hypothesis 2.

In order to test Hypothesis 3, SDT-derived response conditional type 2 ROC curves were plotted in the following way. For each response, we plotted the empirical cumulative distribution for incorrect responses on the x-axis against the cumulative distribution for correct responses that would be expected in an equal-variance SDT model with matching sensitivity and response bias on the y-axis. The difference between the areas of these theoretically derived response conditional type 2 ROC curves was compared against the difference between the true response conditional type 2 ROC curves.

For visualization purposes only, confidence ratings were divided into 20 bins, tailored for each participant to cover their dynamic range of confidence ratings.

For each of the six experiments, Hypotheses 1–4 were tested using a one-tailed t-test at the group level with $\alpha = 0.05$. The summary statistic at the single-subject level was difference in mean confidence between ${S_1}$ and ${S_2}$ responses for Hypothesis 1, difference in area under the response conditional type 2 ROC curves between ${S_1}$ and ${S_2}$ responses ($\Delta AUC$) for Hypothesis 2, difference in $\Delta AUC$ between true confidence distributions and SDT-derived confidence distributions for hypothesis 3, and difference in mean log response time between ${S_1}$ and ${S_2}$ responses for Hypothesis 4.

In addition, a Bayes factor was computed using the BayesFactor R package (Morey *et al.* 2015) and using a Jeffrey–Zellner–Siow (Cauchy) Prior with an rscale parameter of 0.65, representative of the similar standardized effect sizes we observe for Hypotheses 1–4 in our pilot data.

We based our inference on the resulting Bayes factors.

#### Statistical power

Statistical power calculations were performed using the R-pwr packages pwr (Champely 2020) and PowerTOST (Labes *et al.* 2020).

- *Hypothesis 1* (mean confidence): With 106 participants, we had a statistical power of 95% to detect effects of size 0.32, which is less than the standardized effect size we observed for confidence in our pilot sample ($d = 0.66$).
- *Hypothesis 2* (metacognitive asymmetry): With 106 participants, we had a statistical power of 95% to detect effects of size 0.32, which is less than the standardized effect size we observed for metacognitive sensitivity in our pilot sample ($d = 0.73$).
- *Hypothesis 3* (metacognitive asymmetry: control): With 106 participants, we had a statistical power of 95% to detect effects of size 0.32, which is less than the standardized effect size we observed for metacognitive sensitivity, controlling for response bias, in our pilot sample ($d = 0.81$).
- *Hypothesis 4* (response time): With 106 participants, we had a statistical power of 95% to detect effects of size 0.32, which is less than the standardized effect size we observed for response time in our pilot sample ($d = 0.61$).

Finally, in case that the true effect size equals 0, a Bayes factor with our chosen prior for the alternative hypothesis will support the null in 95 out of 100 repetitions and will support the null with a $B{F_{01}}$ higher than 3 in 79 out of 100 repetitions. In a case where the true effect size is sampled from a Cauchy distribution with a scale factor of 0.65, a Bayes factor with our chosen prior for the alternative hypothesis will support the alternative hypothesis in 76 out of 100 repetitions, support the alternative hypothesis with a $B{F_{10}}$ higher than 3 in 70 out of 100 repetitions, and support the null hypothesis with a $B{F_{01}}$ higher than 3 in 15 out of 100 hypotheses (based on an adaptation of simulation code from Lakens 2016).

#### Rejection criteria

Participants were excluded for performing below 60% accuracy, for having extremely fast or slow reaction times (below 250 ms or above 5 s in more than 25% of the trials), and for failing the comprehension check. Finally, for type-2 ROC curves to be generated, some responses must be incorrect, and for them to be informative, some variability in confidence ratings is necessary. Thus, participants who committed less than two of each error type (e.g. mistaking a *Q* of *O* and mistaking an *O* for *Q*) or who reported less than two different confidence levels for each of the two responses were excluded from all analyses.

Trials with response time below 250 ms or above 5 s were excluded.

### Deviations from pre-registration

- *Stimulus used for backward masking*: We planned to use the same stimulus (the letter *Z*) for backward masking in all six experiments. This mask was effective in Experiments 1 and 2, but in Experiment 3, overly high accuracy levels indicated that for these stimuli the mask was not salient enough. For a subset of participants in Experiment 3, an overlay of all seven stimuli from Experiments 3–6 (vertical, tilted, and curved lines, upward-tilted and downward-tilted cubes, and normal and flipped Ns) was used. For the remaining participants and experiments, we used four dollar signs as our mask. See Fig. 2 for depictions of the three masks.
- *Rejection criteria*: In our pre-registration, we explain that informative response conditional type 2 ROC curves can only be generated if participants make errors. When analyzing the data, we came to realize that an additional prerequisite for response conditional type 2 ROC curves to be informative is that the variance in confidence ratings is higher than zero, otherwise the curve is diagonal. We therefore required that participants report at least two different confidence levels for each response. Participants who did not meet this additional criterion were excluded from all analyses.
- *Monetary compensation*: For some of the experiments, we noticed that participants completed the experiment more quickly than what we had originally estimated. We therefore reduced our offered payment for some of the experiments, while maintaining a median hourly wage of £6 or higher.

## Results

**Figure 3.**
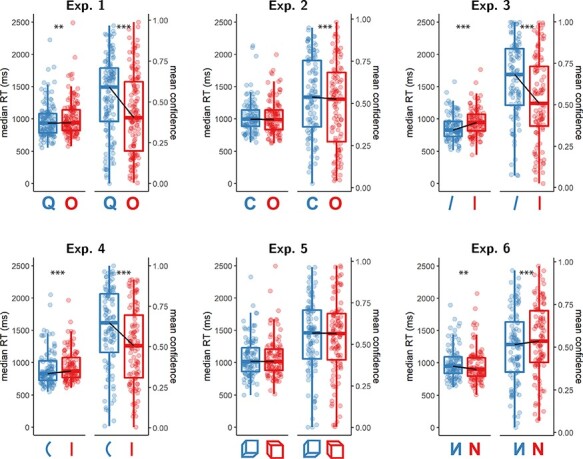
Reaction time and confidence distributions for Experiments 1–6. Box edges and central lines represent the 25, 50, and 75 quantiles. Whiskers cover data points within four inter-quartile ranges around the median. Black lines connect the median values for the two responses. Stars represent significance in a two-sided t-test: **p < 0.01, ***p < 0.001

**Figure 4.**
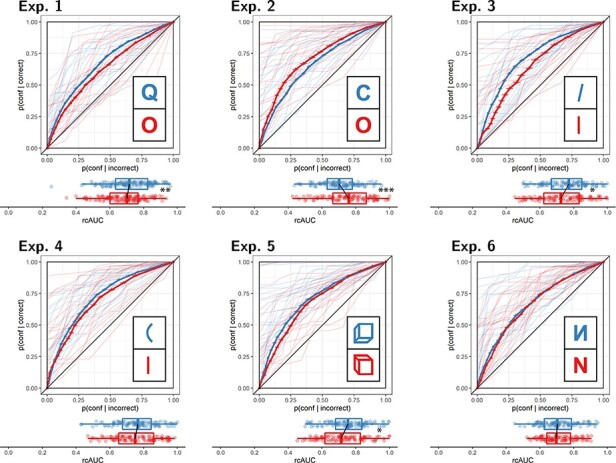
Response conditional type 2 ROC curves for Experiments 1–6. The area under the curve is a measure of metacognitive sensitivity. Error bars stand for the standard error of the mean. For illustration, the curves of the first 20 participants of each experiment are plotted in low opacity. Below each ROC: distributions of the area under the curve for the two responses, across participants. Same conventions as in Fig. 3. Stars represent significance in a two-sided t-test: *p < 0.05, **p < 0.01, ***p < 0.001

**Figure 5.**
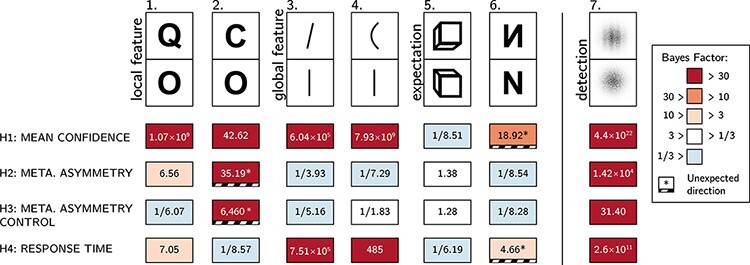
Summary of results from Experiments 1–6 and exploratory Experiment 7. Rows correspond to our four pre-registered hypotheses: a difference in confidence, a difference in metacognitive sensitivity, a difference in metacognitive sensitivity when controlling for response and confidence bias, and a difference in response times

A summary of the results from all six experiments is available in the “Experiments 1–6: summary” and in Figs. 3–5.

### Experiment 1: *Q* versus *O*

In Experiment 1, we examined discrimination judgments between the two letters *Q* and *O*. Based on a search asymmetry for these letters (*Q*s are found faster than *O*s than vice versa; Treisman and Souther 1985), we hypothesized that a similar asymmetry would emerge in subjective confidence judgments, such that metacognitive sensitivity for *Q* responses will be higher than for *O* responses. We used the letter *Z* as our backward mask.

Two hundred and five participants were recruited from Prolific for Experiment 1.

Median completion time was 13.12 minutes. Mean accuracy was 0.74. Participants reported seeing an *O* on 47% of trials. In a deviation from our pre-registration, we excluded nine participants for having zero variance in their confidence ratings for at least one of the two responses (see the “Deviations from pre-registration” section). Overall, we excluded 71 participants based on our exclusion criteria, leaving 134 participants for the main analysis. Due to a technical error in data collection, this figure is higher than that specified in our preregistration document (*n* = 106). Going forward, only data from included participants are analyzed.

Mean accuracy among the included participants was $M = 0.74$, 95% CI $[0.73$, $0.75]$. Mean SOA in the last trial was $M = 47.50$, 95% CI $[39.39$, $55.61]$. Participants showed no consistent bias in their responses (quantified as the probability of a *Q* response minus 0.5; $M = 0.02$, 95% CI $[0.00$, $0.04]$). On a scale of 0–1, mean confidence level was $M = 0.49$, 95% CI $[0.45$, $0.53]$. Confidence was higher for correct than for incorrect responses (${M_d} = 0.15$, 95% CI $[0.13$, $0.17]$, $t\left( {133} \right) = 14.85$, *p* < 0.001).

*Hypothesis 1:* In line with our hypothesis, confidence was generally higher for *Q* (feature present) responses than for *O* (feature absent) responses ($t\left( {133} \right) = 7.52$, *p* < 0.001; Cohen’s *d* = 0.65; BF_10_$ = 1.07 \times {10^9}$; see Fig. 3, panel 1).

*Hypothesis 2:* In order to measure metacognitive asymmetry, we extracted the response conditional type 2 ROC (rc-ROC) curves for the two responses (*Q* and *O*) in the discrimination task. This was done by plotting the cumulative distribution of confidence ratings (high to low) for correct responses against the same distribution for incorrect responses. The auROC_2_ was then taken as a measure of metacognitive sensitivity (Kanai *et al.* 2010; Meuwese *et al.* 2014). In line with our hypothesis, auROC_2_ for *Q* responses ($M = 0.72$, 95% CI $[0.70$, $0.74]$) was higher than for *O* responses ($M = 0.68$, 95% CI $[0.66$, $0.70]$; $t\left( {133} \right) = 2.96$, $p = .002$; Cohen’s *d* = 0.26; BF_10_$= 6.56$; see Fig. 4, panel 1), similar to the documented metacognitive asymmetry for detection judgments.

*Hypothesis 3:* Metacognitive asymmetry was not significantly higher than what is expected based on an equal-variance SDT model with the same response bias and sensitivity as the subjects ($t\left( {133} \right) = 0.97$, $p = .167$; Cohen’s *d* = 0.08). A Bayes factor indicated that our results are more likely under a model that assumes no additional metacognitive asymmetry (BF_01_$= 6.07$).

*Hypothesis 4:* In line with our hypothesis, *Q* responses were faster on average than *O* responses by 37 ms ($t\left( {133} \right) = - 2.99$, $p = .002$; Cohen’s *d* = 0.26; BF_10_$= 7.05$; see Fig. 3, panel 1).

In summary, in Experiment 1, we found that *Q* responses were faster and accompanied by higher subjective confidence, in line with a processing advantage for feature-presence. Metacognitive asymmetry however did not go beyond what is expected from an equal-variance SDT model for these stimuli, taking into account response biases.

### Experiment 2: *C* versus *O*

In Experiment 2, we examined discrimination judgments between the two letters *C* and *O*. Based on a search asymmetry for these letters (*C*s are found faster among *O*s than vice versa; Treisman and Souther 1985; Treisman and Gormican 1988; Takeda and Yagi 2000), we hypothesized that a similar asymmetry would emerge in subjective confidence judgments, such that metacognitive sensitivity for perceiving a *C* will be higher than for perceiving an *O*. We used the letter *Z* as our backward mask.

One hundred and forty-three participants were recruited from Prolific for Experiment 2.

Median completion time was 12.80 minutes. Mean accuracy was 0.75, and participants reported seeing an *O* on 43% of trials. In a deviation from our pre-registration, we excluded eight participants for having zero variance in their confidence ratings for at least one of the two responses (see the “Deviations from pre-registration” section). Overall, we excluded 37 participants, leaving 106 participants for the main analysis. Going forward, only data from included participants are analyzed.

Mean accuracy 47% of trials among included participants was $M = 0.74$, 95% CI $[0.73$, $0.75]$. The mean SOA of the last trial was $M = 40.18$, 95% CI $[34.37$, $46.00]$. Participants showed a consistent bias toward reporting a *C* rather than an *O* ($M = 0.07$, 95% CI $[0.05$, $0.08]$). On a scale of 0–1, mean confidence level was $M = 0.52$, 95% CI $[0.48$, $0.56]$. Confidence was higher for correct than for incorrect responses (${M_d} = 0.17$, 95% CI $[0.15$, $0.19]$, $t\left( {105} \right) = 15.05$, $p \lt .001$).

*Hypothesis 1:* In line with our hypothesis, confidence was generally higher for *C* (feature present) responses than for *O* (feature absent) responses (${M_d} = 0.05$, 95% CI $[0.03$, $\infty )$, $t\left( {105} \right) = 3.59$, $p \lt .001$; Cohen’s *d* = 0.35; BF_10_$= 42.62$; see Fig. 3, panel 2).

*Hypothesis 2:* Opposite to our prediction, auROC_2_ for *C* responses ($M = 0.70$, 95% CI $[0.68$, $0.72]$) was lower than for *O* responses ($M = 0.75$, 95% CI $[0.73$, $0.78]$; $t\left( {105} \right) = - 3.53$, $p \gt .999$; Cohen’s *d* = 0.34; see Fig. 4, panel 2). Bayes factor strongly supported the alternative (BF_10_$= 35.19$). Note that our prior on effect sizes was symmetric around zero, such that support for the alternative is obtained for negative, as well as positive effects.

*Hypothesis 3:* Metacognitive sensitivity for *C* responses was still higher than for *O* responses after controlling for bias (Cohen’s *d* = 0.49; BF_10_$= 6.46 \times {10^3}$).

*Hypothesis 4:* Contrary to our hypothesis, response times for *C* and for *O* responses were highly similar, with a median difference of 6 ms ($t\left( {105} \right) = 0.01$, $p = .504$; Cohen’s *d* = 0.00; BF_01_$= 8.57$; see Fig. 3, panel 2).

In summary, in Experiment 2, we found a dissociation between our two confidence-related measures. As we hypothesized, participants were generally more confident in their *C* (feature present) responses, but their metacognitive sensitivity was higher following *O* (feature absent) responses. We found no reliable difference in response times between these two responses.

### Experiment 3: tilted versus vertical lines

In Experiment 3, we examined discrimination judgments between tilted and vertical lines. Based on a search asymmetry for these stimuli (tilted lines are found faster among vertical lines than vice versa; Treisman and Gormican 1988), we hypothesized that a similar asymmetry would emerge in subjective confidence judgments, such that metacognitive sensitivity for perceiving a tilted line will be higher than for perceiving a vertical line. As described in the “Deviations from pre-registration” section, overly high accuracy in the first few participants led us to change our masking stimulus, first to an overlay of all stimuli and then to four dollar signs. We present here the combined results from these last two cohorts of participants (94 and 210 participants, respectively). The results were qualitatively similar in the two cohorts.

Three hundred and four participants were recruited from Prolific for Experiment 3. Due to shorter than expected completion times in the first 94 participants, the remaining participants were paid £1.25, equivalent to an hourly wage of £6.

Median completion time was 12.43 minutes. Mean accuracy was 0.86, and participants reported seeing a vertical line on 44% of trials. In a deviation from our pre-registration, we excluded 14 participants for having zero variance in their confidence ratings for at least one of the two responses (see the “Deviations from pre-registration” section). Overall, we excluded 198 participants, leaving 106 participants for the main analysis. Going forward, only data from included participants are analyzed.

Mean accuracy 47% of trials among included participants was $M = 0.79$, 95% CI $[0.78$, $0.81]$. The mean SOA of the last trial was $M = 30.83$, 95% CI $[25.99$, $35.68]$. Participants showed a consistent bias toward reporting a tilted rather than a vertical line ($M = 0.06$, 95% CI $[0.04$, $0.08]$). On a scale of 0–1, mean confidence level was $M = 0.61$, 95% CI $[0.56$, $0.65]$. Confidence was higher for correct than for incorrect responses (${M_d} = 0.18$, 95% CI $[0.15$, $0.20]$, $t\left( {105} \right) = 13.42$, $p \lt .001$).

*Hypothesis 1:* In line with our hypothesis, confidence was generally higher for tilted lines (feature present) responses than for vertical lines (feature absent) responses (${M_d} = 0.12$, 95% CI $[0.09$, $\infty )$, $t\left( {105} \right) = 7.18$, $p \lt .001$; Cohen’s *d* = 0.70; BF_10_$= 8.89 \times {10^7}$; see Fig. 3, panel 3).

*Hypothesis 2:* Contrary to our prediction, Bayes factor analysis did not provide evidence for or against a difference in auROC_2_ between reports of seeing a tilted line ($M = 0.76$, 95% CI $[0.74$, $0.78]$) and reports of seeing a vertical line ($M = 0.73$, 95% CI $[0.70$, $0.75]$; Cohen’s *d* = 0.18; BF_01_$= 1.59$; see Fig. 4, panel 3.). A difference in metacognitive sensitivity was however significant in a standard t-test ($t\left( {105} \right) = 1.88$, $p = .031$). With a sample size of 106, a one-tailed t-test is significant for observed effect sizes of 0.16 standard deviations or higher. In contrast, for our choice of a scale factor, a Bayes factor is higher than 3 for observed standardized effect sizes of $0.26$ standard deviations or higher. Effect sizes that fall between 0.16 and $0.26$ are then significant in a t-test, with no conclusive evidence in a Bayes factor analysis. A robustness region analysis revealed that no scale factor would have led to the conclusion that auROC_2_s for the two responses are different with $B{F_{10}} \gt 3$. See Supplementary Fig. S1 for a full Robustness Region plot (Dienes 2019).

*Hypothesis 3:* A Bayes factor analysis did not provide evidence for or against metacognitive asymmetry when controlling for response bias and sensitivity ($t\left( {105} \right) = - 0.70$, $p = .759$; Cohen’s *d* = 0.07; BF_10_$= 6.74$).

*Hypothesis 4:* In line with our hypothesis, response times for “tilted” responses were faster than response times for “vertical” responses, with a median difference of 68 ms ($t\left( {105} \right) = - 5.82$, $p \lt .001$; Cohen’s *d* = 0.56; BF_10_$= 1.83 \times {10^5}$; see Fig. 3, panel 3).

In summary, in Experiment 3, we found that “tilted” (feature present) responses were faster and accompanied by higher subjective confidence than “vertical” (feature absent) responses, with no difference in metacognitive sensitivity between the two responses.

### Experiment 4: curved versus straight lines

In Experiment 4, we examined discrimination judgments between curved and vertical lines. Based on a search asymmetry for these stimuli (curved lines are found faster among vertical lines than vice versa; Treisman and Gormican 1988), we hypothesized that a similar asymmetry would emerge in subjective confidence judgments, such that metacognitive sensitivity for perceiving a tilted line will be higher than for perceiving a vertical line. We used four dollar signs ($$$$) as our mask.

Two hundred and eleven participants were recruited from Prolific for Experiment 4. Due to shorter than expected completion times in previous experiments, participants were paid £1.25, equivalent to an hourly wage of £6.

Median completion time was 12.08 minutes. Mean accuracy was 0.84, and participants reported seeing a straight line on 44% of trials. In a deviation from our pre-registration, we excluded 11 participants for having zero variance in their confidence ratings for at least one of the two responses (see the “Deviations from pre-registration” section). Overall, we excluded 104 participants, leaving 107 participants for the main analysis. Going forward, only data from included participants are analyzed.

Mean accuracy among included participants was $M = 0.79$, 95% CI $[0.77$, $0.80]$. The mean SOA of the last trial was $M = 28.01$, 95% CI $[24.22$, $31.79]$. Participants showed a consistent bias toward reporting a curved rather than a vertical line ($M = 0.06$, 95% CI $[0.04$, $0.07]$). On a scale of 0–1, mean confidence level was $M = 0.57$, 95% CI $[0.53$, $0.61]$. Confidence was higher for correct than for incorrect responses (${M_d} = 0.21$, 95% CI $[0.18$, $0.24]$, $t\left( {106} \right) = 14.96$, $p \lt .001$).

*Hypothesis 1:* In line with our hypothesis, confidence was generally higher for curved lines (feature present) responses than for straight lines (feature absent) responses (${M_d} = 0.12$, 95% CI $[0.09$, $\infty )$, $t\left( {106} \right) = 8.25$, $p \lt .001$; Cohen’s *d* = 0.80; BF_10_$= 1.61 \times {10^{10}}$; see Fig. 3, panel 4).

*Hypothesis 2:* Contrary to our prediction, auROC_2_ for reports of seeing a curved line ($M = 0.76$, 95% CI $[0.73$, $0.78]$) was similar to auROC_2_ for reports of seeing a straight line ($M = 0.75$, 95% CI $[0.73$, $0.78]$; $t\left( {106} \right) = 0.30$, $p = .382$; Cohen’s *d* = 0.03; BF_01_$= 8.23$; see Fig. 4, panel 4).

*Hypothesis 3:* (The lack of) metacognitive asymmetry was not different from what would be expected based on an equal-variance SDT model with the same response bias and sensitivity ($t\left( {106} \right) = - 1.93$, $p = .972$; Cohen’s *d* = 0.19; BF_01_$= 1.45$).

*Hypothesis 4:* In line with our hypothesis, response times for “curved” responses were faster than response times for “straight” responses, with a median difference of 51 ms ($t\left( {106} \right) = - 4.36$, $p \lt .001$; Cohen’s *d* = 0.42; BF_10_$= 558.55$; see Fig. 3, panel 4).

In summary, similar to Experiment 3, “curved” (feature-present) responses were faster and accompanied by higher subjective confidence than “straight” (feature absent) responses. However, similar to the results of Experiment 3, here also we did not find a metacognitive asymmetry for these stimuli.

### Experiment 5: upward-tilted versus downward-tilted cubes

In Experiment 5, we examined discrimination judgments between upward-tilted and downward-tilted cubes. Based on a search asymmetry for these stimuli (upward-tilted cubes are found faster among downward-tilted cubes than vice versa, in line with an expectation to see objects on the ground and not floating in space; Von Grünau and Dubé 1994), we hypothesized that a similar asymmetry would emerge in subjective confidence judgments, such that metacognitive sensitivity for perceiving an upward-tilted cube will be higher than for perceiving a downward-tilted cube. We used four dollar signs ($$$$) as our mask.

One hundred and sixty-two participants were recruited from Prolific for Experiment 5.

Median completion time was 13.30 minutes. Mean accuracy was 0.79, and participants reported seeing a downward-tilted cube on 51% of trials. In a deviation from our pre-registration, we excluded 11 participants for having zero variance in their confidence ratings for at least one of the two responses (see the “Deviations from pre-registration” section). Overall, we excluded 56 participants, leaving 106 participants for the main analysis. Going forward, only data from included participants are analyzed.

Mean accuracy among included participants was $M = 0.77$, 95% CI $[0.76$, $0.78]$. The mean SOA of the last trial was $M = 29.51$, 95% CI $[23.20$, $35.81]$. Participants showed no consistent response bias ($M = - 0.01$, 95% CI $[ - 0.03$, $0.00]$). On a scale of 0–1, mean confidence level was $M = 0.55$, 95% CI $[0.51$, $0.59]$. Confidence was higher for correct than for incorrect responses (${M_d} = 0.23$, 95% CI $[0.20$, $0.26]$, $t\left( {105} \right) = 13.89$, $p \lt .001$).

*Hypothesis 1:* Contrary to our hypothesis, confidence was similar for upward-tilted (feature present) responses and downward-tilted (feature absent) responses (${M_d} = 0.00$, 95% CI $[ - 0.02$, $\infty )$, $t\left( {105} \right) = 0.12$, $p = .452$; Cohen’s *d* = 0.01; BF_01_$= 8.51$; see Fig. 3, panel 5).

*Hypothesis 2:* Contrary to our hypothesis, a Bayes factor analysis did not provide evidence for or against a difference in auROC_2_ for reports of seeing an upward-tilted cube ($M = 0.75$, 95% CI $[0.73$, $0.77]$) and reports of seeing a downward-tilted cube ($M = 0.72$, 95% CI $[0.70$, $0.75]$; Cohen’s *d* = 0.22; BF_10_$= 1.38$; see Fig. 4, panel 5). In contrast, a t-test revealed a significant metacognitive asymmetry, with a higher metacognitive sensitivity for perceiving an upward-tilted (default-violating) cube ($t\left( {105} \right) = 2.29$, $p = .012$). See Supplementary Fig. S1 for a full Robustness Region plot (Dienes 2019).

*Hypothesis 3:* (The lack of) metacognitive asymmetry was not different from what would be expected based on an equal-variance SDT model with the same response bias and sensitivity (Cohen’s *d* = 0.22; BF_10_$= 1.28$). Here also, frequentist and Bayesian analyses conflicted, with a t-test revealing a significant metacognitive advantage for upward-tilted (default violating) responses when controlling for bias ($t\left( {105} \right) = 2.25$, $p = .013$).

*Hypothesis 4:* Contrary to our hypothesis, response times for “upward-tilted” responses were similar to response times for “downward-tilted” responses with a median difference of 9 ms ($t\left( {105} \right) = - 0.82$, $p = .207$; Cohen’s *d* = 0.08; BF_01_$= 6.19$; see Fig. 3, panel 5).

In summary, in Experiment 5, we found no sign of processing asymmetry between upward- and downward-tilted cubes in response times and confidence. A significant metacognitive asymmetry was observed when using null-hypothesis significance testing but was not supported by our Bayes factor analysis. In accordance with our pre-registered plan to commit to the Bayes factor analysis in interpreting the results, in what follows we interpret these findings as providing no support for a metacognitive asymmetry for upward- and downward-tilted cubes.

### Experiment 6: flipped versus normal letters

In Experiment 6, we examined discrimination judgments between flipped and normal *N* stimuli. Based on a search asymmetry for these stimuli (flipped *N*s are found faster among normal *N*s than vice versa; Frith 1974; Wang *et al.* 1994), we hypothesized that a similar asymmetry would emerge in subjective confidence judgments, such that metacognitive sensitivity for perceiving a flipped *N* will be higher than for perceiving a normal *N*. We used four dollar signs ($$$$) as our mask.

One hundred and twenty-seven participants were recruited from Prolific for Experiment 6. Due to shorter than expected completion times in previous experiments, participants were paid £1.25, equivalent to an hourly wage of £6.

Median completion time was 12.76 minutes. Mean accuracy was 0.74, and participants reported seeing a normal *N* on 50% of trials. In a deviation from our pre-registration, we excluded four participants for having zero variance in their confidence ratings for at least one of the two responses (see the “Deviations from pre-registration” section). Overall, we excluded 21 participants, leaving 106 participants for the main analysis. Going forward, only data from included participants are analyzed.

Mean accuracy among included participants was $M = 0.73$, 95% CI $[0.72$, $0.74]$. The mean SOA in the last trial was $M = 37.26$, 95% CI $[33.07$, $41.46]$. Participants showed no consistent response bias ($M = 0.00$, 95% CI $[ - 0.02$, $0.02]$). On a scale of 0–1, mean confidence level was $M = 0.53$, 95% CI $[0.49$, $0.57]$. Confidence was higher for correct than for incorrect responses (${M_d} = 0.17$, 95% CI $[0.15$, $0.20]$, $t\left( {105} \right) = 16.45$, $p \lt .001$).

*Hypothesis 1:* Contrary to our hypothesis, confidence was lower for flipped (feature present) responses than for normal (feature absent) responses. This result was in the opposite direction to what we had expected, so was not significant in a one-tailed t-test (${M_d} = - 0.04$, 95% CI $[ - 0.06$, $\infty )$, $t\left( {105} \right) = - 3.32$, $p = .999$; Cohen’s *d* = 0.32). However, a Bayes factor favored the alternative over the null BF_10_ ($ = 18.92$; see Fig. 3, panel 6).

*Hypothesis 2:* Contrary to our hypothesis, auROC_2_ for reports of seeing a flipped *N* ($M = 0.71$, 95% CI $[0.69$, $0.73]$) was similar to auROC_2_ for reports of seeing a normal *N* ($M = 0.71$, 95% CI $[0.69$, $0.73]$; $t\left( {105} \right) = 0.08$, $p = .468$; Cohen’s *d* = 0.01; BF_01_$= 8.54$; see Fig. 4, panel 6).

*Hypothesis 3:* (The lack of) metacognitive asymmetry was not different from what would be expected based on an equal-variance SDT model with the same response bias and sensitivity ($t\left( {105} \right) = 0.26$, $p = .396$; Cohen’s *d* = 0.03; BF_01_$ = 8.28$).

*Hypothesis 4:* Contrary to our hypothesis, response times for “flipped” responses were slower than response times for “normal” responses, with a median difference of 30 ms ($t\left( {105} \right) = 2.81$, $p = .997$; Cohen’s *d* = 0.27; BF_10_$ = 4.66$; see Fig. 3, panel 6).

In summary, in Experiment 6, we found a difference in response speed and subjective confidence in the opposite direction to what we expected, with a processing advantage for the default-complying stimulus (*N*) compared to the default-violating stimulus (flipped *N*). We found no metacognitive asymmetry for these stimuli.

### Experiments 1–6: summary

Overall, the pattern of results from Experiments 1–6 only partly matched our hypotheses in some cases and stood in direct contrast to them in other cases (see Fig. 5). A reliable metacognitive asymmetry was observed only in Experiment 2, and this asymmetry was in the opposite direction to what we had predicted, with a metacognitive advantage for *O* (feature absent) over *C* (feature present) responses. A metacognitive advantage for reporting *Q* over *O*s (Exp. 1) was not reliably above what is expected based on an equal-variance signal detection model.

For both local and global visual features (Experiments 1–4), we observed differences in mean confidence and response times that were consistent with our hypothesis of a processing advantage for the representation of the presence compared to the absence of visual features. In Experiments 5 and 6, we tested more abstract expectation violations. In Experiment 5, discrimination between upward-tilted and downward-tilted cubes showed no asymmetry in response time and confidence. In Experiment 6, participants were less confident and slower in their reports of seeing a flipped *N*, contrary to our prediction that default-violating signals should be easier to perceive. We found no evidence for or against a difference in metacognitive sensitivity in either of the experiments.

### Experiment 7 (exploratory): grating versus noise

**Figure 6.**
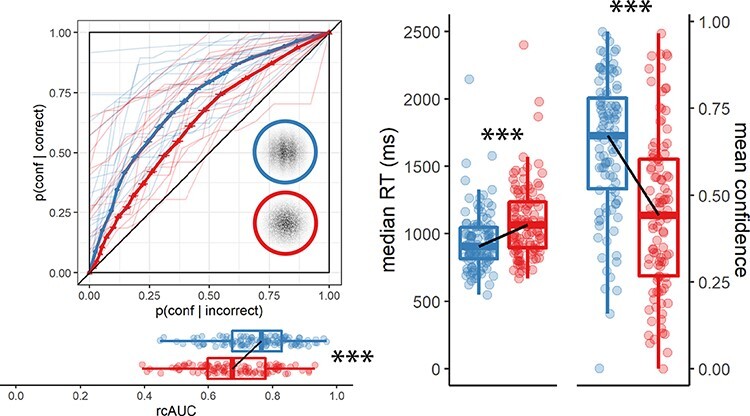
Response conditional type 2 ROC curves (left panel) and confidence and reaction time distributions (right panel) for Experiment 7 (detection positive control). The structure of this figure is similar to Figures 3 and 4: ***p < 0.001

Results from Experiments 1–6 revealed that search asymmetry is not always accompanied by an asymmetry in metacognitive sensitivity. Given that we did not observe a true metacognitive asymmetry in the expected direction for any of our stimulus pairs, we were concerned that our experimental design may have been unsuitable for detecting classical metacognitive asymmetries in detection, for example, due to an insufficient number of trials, the masking procedure or the confidence report scheme. As a positive control, we collected data for an additional experiment that more closely resembled typical detection experiments. In this experiment, participants discriminated between two stimuli: random noise and a noisy grating (presented to participants as a “zebra” stimulus; see Fig. 6). In a previous lab-based study, similar stimuli produced a robust metacognitive asymmetry between target-absent (noise) and target-present (noisy grating) responses (Mazor *et al.* 2020). We used black and white concentric circles as a mask. Apart from the choice of stimuli and mask, the procedure was identical to that of our pre-registered experiments.

One hundred and twenty-seven participants were recruited from Prolific for exploratory Experiment 7. For this positive control, all four hypotheses were fulfilled.

Median completion time was 10.70 minutes. Mean accuracy was 0.73, and participants reported seeing a grating on 48% of trials. Overall, we excluded 36 participants, leaving 105 participants for the main analysis. Going forward, only data from included participants are analyzed.

Mean accuracy among included participants was $M = 0.76$, 95% CI $[0.74$, $0.77]$. The mean SOA of the last trial was $M = 53.87$, 95% CI $[38.85$, $68.89]$. Participants showed no consistent response bias ($M = 0.01$, 95% CI $[0.00$, $0.03]$). On a scale of 0–1, mean confidence level was $M = 0.55$, 95% CI $[0.51$, $0.59]$. Confidence was higher for correct than for incorrect responses (${M_d} = 0.15$, 95% CI $[0.13$, $0.17]$, $t\left( {104} \right) = 12.58$, $p \lt .001$).

*Hypothesis 1:* In line with our hypothesis, confidence was higher for reports of target presence than for reports of target absence (${M_d} = 0.20$, 95% CI $[0.17$, $\infty )$, $t\left( {104} \right) = 14.07$, $p \lt .001$; Cohen’s *d* = 1.37; BF_10_$ = 4.39 \times {10^{22}}$; see Fig. 6, right panel).

*Hypothesis 2:* In line with our hypothesis, auROC_2_ for reports of target presence ($M = 0.75$, 95% CI $[0.73$, $0.77]$) was higher than for reports of target absence ($M = 0.68$, 95% CI $[0.66$, $0.70]$; $t\left( {104} \right) = 5.20$, $p \lt .001$; Cohen’s *d* = 0.51; BF_10_$ = 1.42 \times {10^4}$; see Fig. 6, left panel).

*Hypothesis 3:* In line with our hypothesis, this metacognitive asymmetry was stronger than what is expected based on an equal-variance SDT model with the same response bias and sensitivity ($t\left( {104} \right) = 3.49$, $p \lt .001$; Cohen’s *d* = 0.34; BF_10_$ = 31.40$).

*Hypothesis 4:* In line with our hypothesis, reports of target presence were faster than reports of target absence, with a median difference of 124 ms ($t\left( {104} \right) = - 8.84$, $p \lt .001$; Cohen’s *d* = 0.86; BF_10_$ = 2.63 \times {10^{11}}$; see Fig. 6, right panel).

### Exploratory analysis

#### zROC analysis

In a signal-detection framework, metacognitive asymmetry appears when the signal distribution has both a higher mean and a higher variance than that of the noise distribution. This unequal variance setting produces a higher metacognitive sensitivity for judgments of signal presence, compared to judgments of signal absence. A direct measure for the ratio between the variances of the two distributions is the slope of the *type-1 zROC curve*. A zROC curve is constructed by applying the inverse of the normal cumulative density function to false alarm and hit rates for different confidence thresholds. The slope of the zROC curve equals 1 exactly when the variance of the signal and noise distributions is equal. In detection experiments, the slope is often shallower than 1, indicating a wider signal distribution. Indeed, in our positive control experiment (Experiment 7), the median zROC slope was 0.86 and significantly shallower than 1 ($t\left( {103} \right) = - 5.08$, $p \lt .001$ for a t-test on the log-slope against zero). Measuring the slope of the zROC curve in our six pre-registered experiments, we asked whether our “feature-present” distributions had higher variance than our “feature-absent” distributions. We used the standardized effect size obtained from Experiment 7 as a scaling factor for the prior distribution over effect sizes, reflecting a belief that a difference in slopes should be similar in magnitude to what is observed in a detection task. zROC slopes were numerically shallower than one in Experiments 1 (*Q* versus *O*; median slope = 0.95), 3 (line tilt; median slope = 0.94), 4 (line curvature; 0.97) and 5 (cube orientation; 0.95). This was significant only in Experiment 5 ($t\left( {101} \right) = - 2.09$, $p = .039$). In agreement with the results of our rc-ROC analysis, the zROC slope in Experiment 2 (‘C’ versus ‘O’) was significantly higher than 1, suggesting that the representation of the letter ‘O’ was more variable than that of the letter ‘C’ (median slope = 1.09; $t\left( {104} \right) = 2.29$, $p = .024$). A Bayes factor analysis did not provide support for or against the null hypothesis for any of the six experiments (all Bayes factors between 1/3 and 3).

Previous studies reported similar variance structures for these stimuli when presented in visual search arrays. For example, confidence in a vertical/tilted visual search task revealed a higher variance in the representation of tilted (feature positive) compared to vertical (feature negative) stimuli (Vincent 2011). Similarly, reverse correlation analysis revealed a higher variance in the representation of *Q* (feature positive) compared to *O* (feature negative) stimuli (Saiki 2008). Finally, and in agreement with our results, variance in the representation of *O* (feature negative) was found to be higher than in the representation of *C* (feature positive) (Dosher *et al.* 2004). Note that for the case of line tilt and *Q* versus *O*, finding a high-variance target among low-variance distractors is easier than finding a low-variance target among high-variance distractors. However, the opposite is true for *C* versus *O*, where a low-variance target (*C*) renders the search easier. This last observation challenges the suggestion that variance structure is the determining factor for visual search asymmetries (Treisman and Gormican 1988; Dosher *et al.* 2004; Saiki 2008; Vincent 2011).

#### Inter-subject correlations

**Figure 7.**
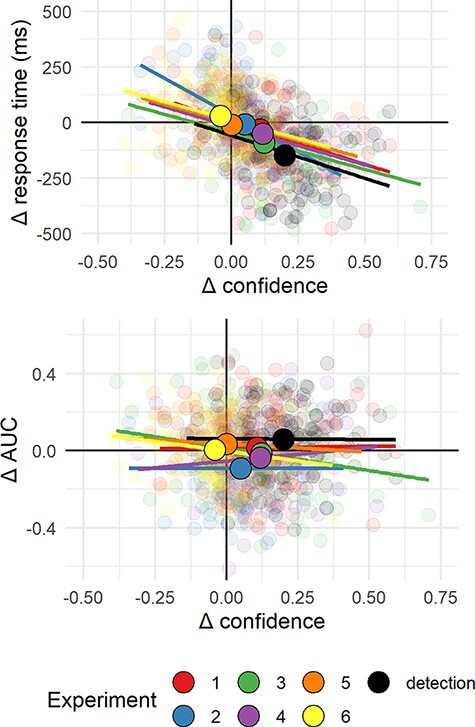
Upper panel: Difference in mean confidence between S1 and S2 responses plotted against difference in mean response time between S1 and S2 responses across the seven experiments. Lower panel: Difference in mean confidence between S1 and S2 responses plotted against difference in metacognitive sensitivity, controlling for response bias, across the seven experiments. Semi-transparent circles represent individual subjects. Opaque circles are the means for each of the seven experiments, across participants. Lines indicate the best-fitting linear regression line for Experiments 1–7

Across experiments, asymmetry in mean confidence (Hypothesis 1) and in response time (RT; Hypothesis 4) was mostly aligned. This is consistent with previous reports of a negative correlation between response times and confidence across trials within participants (Henmon 1911; Pleskac and Busemeyer 2010; Moran *et al.* 2015; Calder-Travis *et al.* 2020). To test if this was the case across participants too, and not only across experiments, we fitted a mixed-effects regression model to data from all seven experiments with experiment as a random effect ($\Delta RT \sim \Delta conf + (1 + \Delta conf|exp)$). The association between confidence and RT effects was significant in this model ($p \lt 0.001$; see Fig. 7; upper panel). In contrast, metacognitive asymmetry (difference between the area under the response conditional type 2 ROC curves, controlling for response bias) was not significantly associated with asymmetry in either confidence ratings ($p = 0.41$; see Fig. 7; lower panel) or reaction time ($p = 0.54$).

## Discussion

In perceptual detection, judgments about the presence or absence of a target stimulus differ in several ways. First, participants are more confident in stimulus presence than in stimulus absence (e.g. Meuwese *et al.* 2014; Kellij *et al.* 2021). Second, confidence ratings in judgments of stimulus presence are more aligned with objective accuracy (Meuwese *et al.* 2014; Kellij *et al.* 2021; Mazor *et al.* 2020). Finally, participants are faster to report stimulus presence (Mazor *et al.* 2020). In our positive control detection experiment (Experiment 7), we replicated these detection asymmetries. We found a mean difference of 20% confidence between decisions about the presence or absence of a grating, a metacognitive asymmetry of 0.07 in area under the curve (AUC) units (ranging from 0 to 1), and a median difference of 124 ms in response time between reports of target presence and absence.

In six pre-registered experiments, we focused on these three behavioral signatures of decisions about the presence and absence of a stimulus and asked whether they extend to discrimination tasks where stimuli are distinct in the presence or absence of sub-stimulus features such as the presence of an additional line in a letter, the curvature of a line, or, more abstractly, the presence of a surprising default-violating signal. Our six stimulus pairs have been shown in previous studies to produce asymmetries in visual search, potentially reflecting differences in the processing of presences and absences of visual features and of default-complying versus default-violating stimuli. If detection asymmetries also reflect differences in the abstract processing of presences and absences, or of default-complying versus default-violating sensory input, one would expect to find detection-like asymmetries in response time, confidence, and metacognitive sensitivity for discrimination between stimuli that produce asymmetries in a visual search task.

Starting from the end, Experiments 5 and 6 provide evidence against the proposal that asymmetries in confidence, reaction time, and metacognitive sensitivity emerge for default-violating signals at all levels of representation. Stimulus pairs in Experiments 5 (cube orientation) and 6 (letter inversion) produced response conditional type 2 ROC curves that were more consistent with the absence of metacognitive asymmetry than with our prior distribution on effect sizes (see the “Dependent variables and analysis plan” section for the specifics of our Bayesian hypothesis testing, including our prior on effect sizes). Given that these stimuli have been shown to produce reliable asymmetries in visual search (Frith 1974; Von Grünau and Dubé 1994; Wang *et al.* 1994; Malinowski and Hübner 2001; Shen and Reingold 2001), we can safely conclude that not all default violations that produce an asymmetry in visual search also produce an asymmetry in metacognitive sensitivity.

Moreover, in Experiment 6, default-complying *N* responses were faster, and accompanied by higher levels of subjective confidence, than default-violating flipped-*N* responses. This is in contrast to our prediction of a processing advantage for default-violating signals and in line with previous reports of a processing advantage for familiar over unfamiliar stimuli in the context of face perception and reading. For example, in a breaking continuous flash suppression paradigm, inverted faces took longer to break into awareness than upright faces (Stein and Peelen 2021). A similar processing advantage for familiar stimuli has been documented for the perception of words (Albonico *et al.* 2018) and Chinese letters (Xue *et al.* 2006). One possibility is that the perception of highly familiar stimuli such as letters and faces is supported by specific expert brain systems, affording a processing advantage beyond the general superior processing of default-violating signals (Yovel and Kanwisher 2005; Xue *et al.* 2006). Indeed, Experiment 6 was the only experiment in which we observed a processing advantage for familiar over unfamiliar stimuli.

Next, in Experiments 3 and 4, we looked at two features that have a global effect on stimulus appearance: tilt and curvature. Based on visual search asymmetries, Treisman and Gormican (1988) proposed that tilt and curvature are represented as positive features in the visual system. This takes us one step closer to typical detection experiments: participants now detect the presence or absence of a basic visual feature. In agreement with our Hypotheses 1 and 4, participants were more confident in identifying tilted and curved lines (mean differences of 0.12 and 0.12 on a 0–1 confidence scale) and were faster in giving these responses (mean differences of 67.67 and 50.57 ms). However, we did not find evidence for or against a metacognitive asymmetry for these global visual features.

Our strongest candidate for a stimulus pair for which we expected to find a presence-absence asymmetry was *Q* versus *O* (Experiment 1). The difference between these two letters is the presence of an additional line stroke: a concrete stimulus part that is localized in space and is independent of the rest of the stimulus. Theoretically, participants could approach this task as a detection task: ignore the common denominator (*O*) and focus on the presence or absence of the distinctive feature (“,”). As we hypothesized, participants were more confident in their *Q* responses (mean difference of 0.11 on a 0–1 confidence scale). Participants were also faster in their *Q* responses (median difference of 37 ms). However, unlike a stimulus-level detection, a small difference of 0.04 units in the area under the response conditional ROC curves was not different than what is expected based on a null SDT model.

Finally, In Experiment 2, we looked at discrimination between *C* and *O*s based on evidence from the visual search that open edges are represented as a positive feature in the visual system (Treisman and Souther 1985; Treisman and Gormican 1988; Takeda and Yagi 2000). As we hypothesized, *C* responses were accompanied by higher levels of subjective confidence (mean difference of 0.05 on a 0–1 confidence scale) and were delivered faster than *O* responses (with a modest but significant difference of 6 ms between the two responses). However, in striking contrast to our original hypothesis, metacognitive sensitivity was lower for *C* responses (mean difference of 0.05 AUC units), even when controlling for response bias. This result strongly supports different underlying mechanisms behind search and metacognitive asymmetries. Furthermore, the results of Experiment 2 suggest distinct factors mediate the processing advantage for presence over absence (as reflected in shorter response times and higher confidence for *C* responses) and the metacognitive asymmetry between presence and absence (as reflected in improved metacognitive sensitivity for *O* responses).

*C* and *O* are unique in that the difference between them corresponds to two contrasting notions of presence and absence. On the one hand, *C* is marked by the presence of one additional feature—open edges (Treisman and Souther 1985; Treisman and Gormican 1988). On the other hand, it is marked by the absence of a piece: there is simply less of it relative to *O*. These two notions of presence and absence are typically coupled in detection. For example, the presence of a grating on a screen corresponds to the presence of additional features (such as orientation, contrast, and phase) as well as of more “visual stuff,” relative to the blank background. A compelling interpretation of the results of Experiment 2 is that it is the presence or absence of visual features such as open edges that is driving the difference in confidence and response time, whereas a more quantitative notion of presence or absence (the amount of “visual stuff” presented) is driving the metacognitive asymmetry between these two responses. We note however that based on this interpretation, we would expect a metacognitive sensitivity to operate also in Experiment 1, where *O* is missing a piece relative to *Q*. As described above, Experiment 1 provided no evidence for such a metacognitive asymmetry beyond what is expected from an equal-variance signal-detection model.

Notably, not one of the six pre-registered experiments produced a metacognitive asymmetry in the expected direction. This was in contrast to Experiment 7 (grating versus noise), where metacognitive sensitivity for reporting noise was lower than for reporting a noisy grating (with a difference of 0.07 auROC_2_ units, BF_10_$= 31.40$). Positive control Experiment 7 was also the only experiment in which we found a higher variance for stimulus S1 than for stimulus S2 (with a median variance ratio of 0.86). These two observations are likely to be related: across participants, metacognitive asymmetry and variance ratio were highly correlated ($r = .64$, 95% CI $[.51$, $.74]$, $t\left( {102} \right) = 8.42$, $p \lt .001$). Indeed, previous theoretical work has pointed out that response-dependent asymmetries in metacognition may be driven by an underlying unequal-variance SDT model and, vice versa, that findings of unequal variance might be due to a response-dependent metacognitive asymmetry. These two perspectives are interchangeable (Maniscalco and Lau 2014). However, a correlation between metacognitive asymmetry and variance structure, both estimated from confidence ratings, is not a satisfactory answer for why noise and gratings should exhibit a unique asymmetry in metacognitive sensitivity or in a variance structure. More theoretical and experimental work is needed to identify the sources of this asymmetry, perhaps focusing on the role of stimulus complexity and perceptual uncertainty as potential drivers of this effect.

When interpreting our findings in a broader context, it is useful to note that in all six experiments we used backward masking for controlling the visibility level of our stimuli. Different visibility manipulations have been shown to affect detection metacognitive sensitivity in different ways. For example, whereas metacognitive sensitivity in detection of “no” responses is at chance when backward masking is used, it is significantly higher than chance when the attentional blink is used to control the stimulus visibility (Kanai *et al.* 2010). Similarly, phase scrambling but not attentional blink produces a metacognitive advantage for “yes” responses (Kellij *et al.* 2021). While our positive control (Experiment 7) produced a reliable metacognitive asymmetry between judgments of target presence and absence, it was also the only experiment where stimulus visibility was controlled with low contrast, in addition to backward masking (for the purpose of compatibility with previous experiments; see Fig. 6). Based on our findings alone, we cannot rule out the possibility that using other visibility manipulations may reveal metacognitive asymmetries for the presence or absence of abstract default violations. Furthermore, it is possible that some of the observed asymmetries for low-level features may reflect asymmetries in the joint perception of target stimulus and backward mask, rather than in the perception of the target stimulus by itself (Kahneman 1968; Jannati and Di Lollo 2012).

Together, our findings weigh against our original proposal that metacognitive asymmetries in perceptual detection are a signature of higher-order default reasoning. Unlike search asymmetries that extend to abstract levels of representations such as familiarity (Wang *et al.* 1994; Wolfe 2001) and even social features such as ethnicity and gender (Levin and Angelone 2001; Gandolfo and Downing 2020), metacognitive asymmetries in visual discrimination are grounded in concrete visual processing. Furthermore, we provide evidence for a dissociation between asymmetries in metacognition and in response time and confidence, where the latter is linked to activation of basic feature detectors, for example, of orientation, open ends, or curvature.

## Conclusion

In a set of six experiments, we sought to test the proposal that a metacognitive asymmetry between the representation of stimulus presence and absence is one instance of a more general asymmetry between the representation of default states and default-violating surprises. Our findings provide evidence against this idea. A metacognitive asymmetry was not observed in near-threshold discrimination between stimulus pairs that differ in their alignment with default expectations. This was the case even in pairs that showed substantial asymmetries in response time and overall confidence levels. Results from our pre-registered set of analyses are most consistent with a narrow interpretation of the presence/absence metacognitive asymmetry in visual detection, that is limited to concrete, spatially localized presences. Furthermore, a metacognitive asymmetry between *C*s and *O*s in the opposite direction to what is observed in visual search indicates that different cognitive and perceptual mechanisms underlie these two apparently similar phenomena.

## Supplementary Material

niab025_Supp

## Data Availability

All raw data are fully available on OSF and on the study’s GitHub respository: https://github.com/matanmazor/asymmetry.

## Code availability

All analysis code is openly shared on the study’s GitHub repository: https://github.com/matanmazor/asymmetry. For complete reproducibility, the RMarkdown file used to generate the final version of the manuscript, including the generation of all figures and extraction of all test statistics, is also available on our GitHub repository.
