# Supplementary material for: Stage 2 Registered Report: Metacognitive asymmetries in visual perception
Source: Neurosci Conscious. 2021 Dec 13;2021(1):niab025. doi: 10.1093/nc/niab025 (PMC8524176; doi:10.1093/nc/niab025)
Supplement: niab025_Supp [file niab025_supp.zip › Appendix.docx]

Appendix

## S.1 Robustness Region


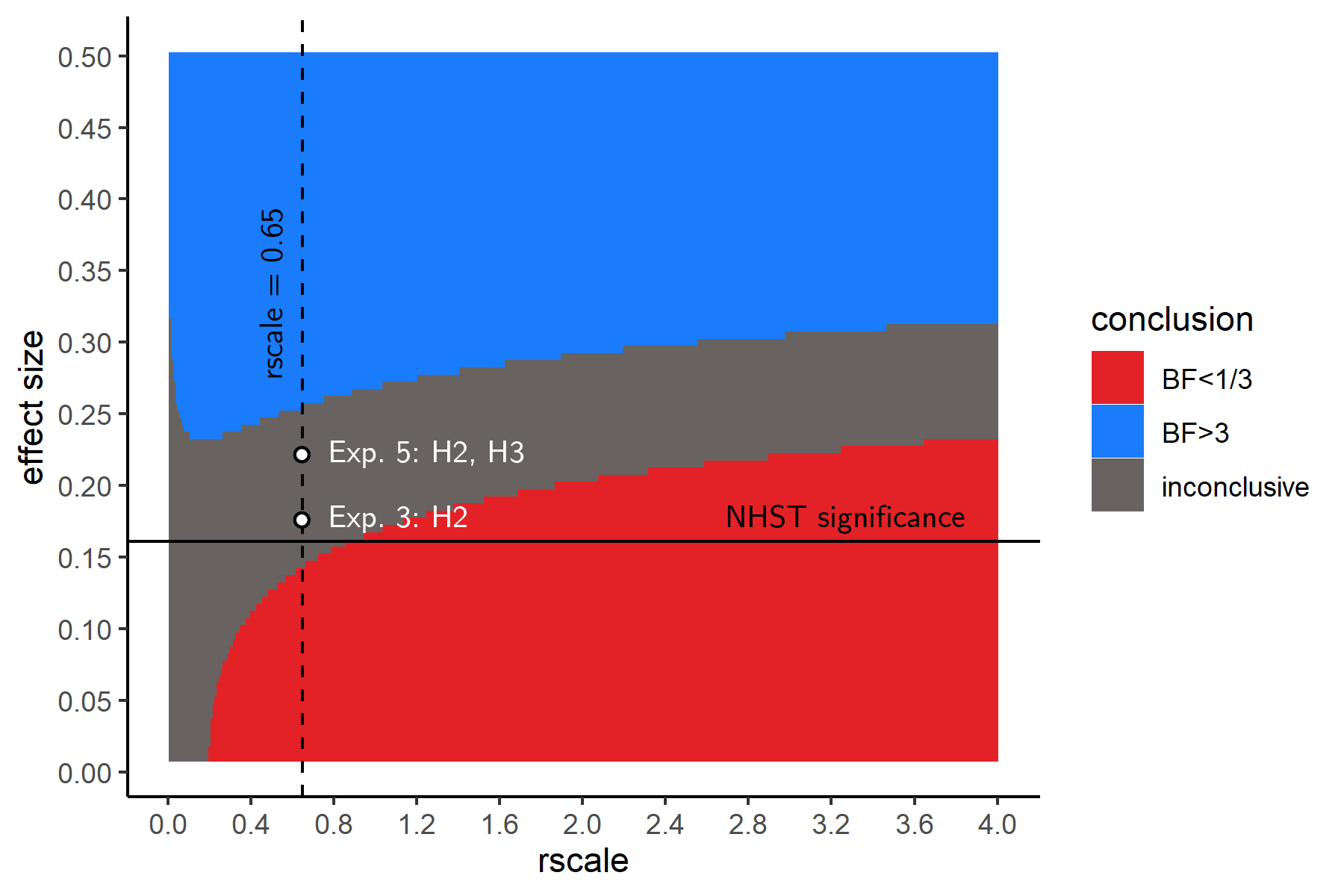


*Figure* *S1.*  A Robustness Region plot, visualizing Bayes Factors for hypothetical effect sizes and scale factors on the prior, for our sample size of 106 participants. Points above the horizontal line are significant in a one-tailed t-test. The dashed line indicates our choice of a scale factor on the prior.
